# LONG-TERM EXPOSURE TO AIR POLLUTION AND RISK OF DEMENTIA AMONG OLDER INDIVIDUALS OF A DANISH NATIONWIDE ANALYSES

**DOI:** 10.1093/geroni/igae098.1252

**Published:** 2024-12-31

**Authors:** Rina So, Zorana Andersen, Youn-Hee Lim, Jiawei Zhang, Stéphane Tuffier, Thomas Cole-Hunter

**Affiliations:** University College London, London, England, United Kingdom; University of Copenhagen, Copenhagen, Sjelland, Denmark; University of Copenhagen, Copenhagen, Hovedstaden, Denmark; University of Copenhagen, Copenhagen, Hovedstaden, Denmark; University of Copenhagen, Copenhagen, Hovedstaden, Denmark; University of Copenhagen, Copenhagen, Hovedstaden, Denmark

## Abstract

The evidence linking air pollution with dementia has been fast growing over the last decade, but who is most susceptible remains rarely explored. Therefore, we aimed to examine the association between long-term exposure air pollution and incidence of dementia and identify the most susceptible groups by age, sex, socioeconomic status (SES), and comorbidities with major chronic diseases. We followed all Danish residents aged 60 years or older on January 1st, 2000 until December 31st, 2018. Annual mean concentrations of fine particulate matter (PM2.5), nitrogen dioxide (NO2), and black carbon (BC) in 2010 (100x100m) were estimated using European-wide hybrid land-use regression models and assigned to the baseline residential address. We used Cox proportional hazard models to evaluate the association between air pollution and dementia incidence, accounting for individual and area-level SES, and introduced an interaction term to test for effect modification by age, sex, income, education, employment, and co-morbidity with cardio-metabolic, respiratory diseases and depression. Of 934,792 subjects, 81,731 developed dementia. We detected statistically significant associations of all three pollutants with incidence of dementia, with hazard ratios (HRs) and 95% confidence intervals (CIs) of 1.14 (1.12-1.16) per 1.9 μg/m3 increase in PM2.5, 1.25 (122-1.28) per 10.2 μg/m3 increase in NO2, and 1.23 (1.20-1.26) per 0.5×10-5/m increase in BC. Associations were stronger in the elderly (≥75 years), stroke patients, unemployed, and those with the lower income or education. In this nationwide analysis in a low-exposure setting in Demark, long-term exposure to air pollution contributes to the development of dementia.

